# Incidence of urinary tract infection in neonates with significant indirect Hyperbilirubinemia of unknown etiology: case-control study

**DOI:** 10.1186/s13052-021-00982-0

**Published:** 2021-02-17

**Authors:** Ahmed Mahrous Kamal Baz, Osama Abd El-Fattah El-Agamy, Ashraf Mohamed Ibrahim

**Affiliations:** 1 Neonatology Unit, Pediatrics department, Kafrelsheikh Faculty of Medicine, Ibrahim Moghazy st. 21, Kafrelsheikh, 33511 Egypt; 2 Kafrelsheikh Faculty of Medicine, Kafrelsheikh, Egypt; 3 Neonatology Unit, Pediatrics department, Tanta Faculty of Medicine, Tanta, Egypt

**Keywords:** Hyperbilirubinemia, Incidence, Neonates, Urinary tract infection

## Abstract

**Background:**

Indirect hyperbilirubinemia is frequently encountered during neonatal period. Although it has different causes, in some cases it can’t be explained. Previous studies have illustrated that jaundice could be a major sign of urinary tract infection (UTI) in neonates.

**Aim of the work:**

We aimed to determine the association between UTI and significant unexplained neonatal indirect hyperbilirubinemia.

**Methods:**

This prospective controlled study was performed on 150 neonates divided in two groups (100 as cases and 50 as controls) to investigate the incidence of UTI in neonates with significant unexplained hyperbilirubinemia. Urine sample was obtained using urine catheterization technique from neonates and full urine analysis was done and cases with pyuria had urine culture to confirm UTI. Immediate renal ultrasonography (USG) was performed for neonates with UTI.

**Results:**

UTI incidence was 11% in cases while none of neonates in control group had UTI with statistical significance between cases and controls (*P* value < 0.05). The most common (36.4%) pathogen was *Escherichia coli*. Posterior urethral valve with mild hydronephrosis was diagnosed in 18.2% of UTI positive patients by renal ultrasonography.

**Conclusion:**

In neonates with unexplained indirect hyperbilirubinemia, UTI should be considered as a pathological cause.

**Supplementary Information:**

The online version contains supplementary material available at 10.1186/s13052-021-00982-0.

## Introduction

Hyperbilirubinemia is frequent in newborns. In some newborns, total serum bilirubin doesn’t reach pathological levels while in others it may exceed physiological levels and requires treatment. Many well-known causes could be detected during routine investigation of neonates with significant hyperbilirubinemia while in others it may remain unexplained [1].

Neonatal jaundice is mainly physiological and 60% of neonates have it. A small number of those neonates have pathological problems like ABO or Rh incompatibility, hepatic impairment, systemic infection or metabolic disease. In neonates with Urinary Tract Infections (UTIs), jaundice could be an early sign [2].

One of the causes of prolonged jaundice is UTI. UTI investigations have been included in routine workup in neonates with prolonged jaundice. There was high incidence of UTI in neonates who developed jaundice after first week of neonatal age in study performed by Garcia and Nager. Ghaemi et al. also observed equal incidence rate of UTI in neonates with prolonged jaundice and neonates with febrile illness with matched ages. On the contrary, it was observed by Omar et al. and Bilgen et al. that asymptomatic neonates with UTI developed jaundice during the first week of neonatal age. Therefore, besides investigating UTI in prolonged jaundice, it was recommended that testing for UTI should be included in the workup of neonates who develop jaundice in early neonatal period [3].

The aim of this study was to detect if UTI is a significant cause of unexplained indirect hyperbilirubinemia in jaundiced neonates who require treatment and should it be included in routine workup of those neonates.

## Methods

This analytical case-control study was conducted on 100 neonates (43 males & 57 females) at NICU in Kafr Elsheikh University Hospital with a gestational age greater than 35 weeks with significant unexplained indirect hyperbilirubinemia as cases and on 50 matched neonates (25 males and 25 females) without hyperbilirubinemia as controls. Cases were recruited during study period from January 2019 till February 2020.

Neonates with hemolytic diseases, glucose-6-phosphate dehydrogenase (G6PD) deficiency, neonatal sepsis, polycythemia, with direct hyperbilirubinemia, hypothyroidism and metabolic diseases were excluded.

All newborns were subjected to careful history taking including mode of delivery, full maternal history, maternal blood grouping for detection of incompatibility, family history of jaundice and history of G6PD in a family member. Full clinical examination was done with performing the following investigations serum total and direct bilirubin, full blood count, reticulocyte index and peripheral blood smear, coomb’s test (direct), C reactive protein (CRP), urea and creatinine, liver functions, G6PD enzyme assay, thyroid function (TSH and free T4). All study participants had a urine sample obtained using urinary catheterization technique under a complete aseptic technique. The urine sample was tested using Multistix 10 for standard urinalysis. The samples were examined microscopically under high power field (HPF) for pyuria. Pyuria was defined as > 5 white blood cell (WBC) per HPF. Patients whom urine analysis results showed pyuria had urine culture to confirm presence of UTI and detect causing organism. Culture was done using CLED agar (cysteine, lactose and electrolyte deficient agar). The culture was considered positive when it detected the presence of at least 50,000 CFUs per mL of a single urinary pathogen. Neonates with confirmed urinary tract infection were investigated by renal ultrasonography to exclude congenital anomalies.

### Statistical analysis

Data were fed to the computer and analyzed using IBM SPSS software package version 20.0***.*** **(**Armonk, NY: IBM Corp**).** The Kolmogorov-Smirnov test was used to verify the normality of distribution Quantitative data were described using range (minimum and maximum), mean, standard deviation, median and interquartile range (IQR). Significance of the obtained results was judged at the 5% level.

The used tests were.

1 - chi-square test

For categorical variables, to compare between different groups.

2 - Fisher’s exact or Monte Carlo correction

Correction for chi-square when quite 20% of the cells have expected count but 5.

3 - Mann Whitney test

For abnormally distributed quantitative variables, to match between two studied groups.

## Results

A sum of 150 neonates presented to the NICU fulfilling criteria of case and control groups. Of the 100 neonates of cases group, 57 were female, and 43 were male. Of the 50 neonates of control group, 25 were males and 25 were females. All males were uncircumcised. As illustrated in **(**Table 1**),** no significant statistical difference between case and control groups was observed regarding demographic data.

**Table 1:** Association between case and control groups according to some demographic data and laboratory values

	Cases (***n*** = 100)	Control (***n*** = 50)	***P*** value
	Mean ± SD		
**Age on admission**	118.80 ± 51.66	119.04 ± 52.66	0.976
**Weight (kg)**	3.13 ± 0.34	3.13 ± 0.34	1.00
**Total bilirubin (mg/dl)**	17.90 ± 1.71	2.18 ± 0.87	< 0.001^*^
**Direct bilirubin (mg/dl)**	0.68 ± 0.34	0.40 ± 0.22	< 0.001^*^
**Hemoglobin (gm/dl)**	15.42 ± 1.42	15.61 ± 1.59	0.478
**Reticulocyte count (%)**	1.70 ± 0.82	1.70 ± 0.82	0.984
**Urea (mg/dl)**	16.73–4.27	16.91–4.29	0.775
**Creatinine (mg/dl)**	0.40–0.22	0.39–0.21	0.732

p: *p* value for comparing between the studied groups

*: statistically significant at *p* ≤ 0.05

Sixty-five percent of case group were exclusively breastfed, 20% were formula fed, and 15% depended on both types of feeding. Fourteen neonates were treated with intensive phototherapy; the rest were treated with conventional phototherapy. None of the studied neonates had fever. Laboratory data of case and control groups are illustrated in (Table 1). UTI was confirmed in eleven neonates of case group while no case in control group had confirmed UTI. The most common microorganisms in urine culture was *Escherichia coli* (36.4%) followed by acitenobacter bacilli (27.3%) (Table 2).

**Table 2:** Microorganisms isolated by urine culture in group A (newborns with indirect hyperbilirubinemia and urinary tract infection confirmed by urine culture) (*n* = 11)

	Number	%
**Gram positive organisms**		
Staphylococcus aureus	1	9.1
Enterococci	1	9.1
**Gram negative organisms**		
Gram Negative Bacilli *E. coli*	4	36.4
Gram Negative Bacilli Acinetobactar	3	27.3
Gram negative bacilli (Klebsiella Pneumonia)	2	18.2

**Fig. 1:**
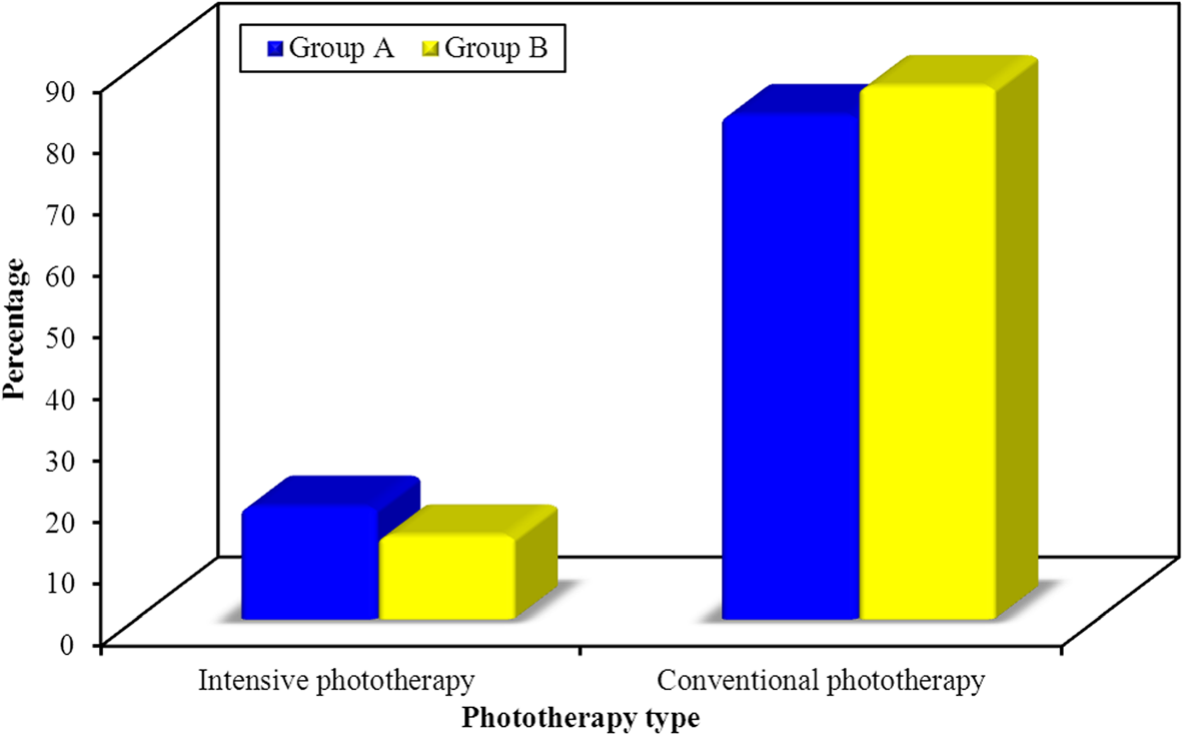
Bar chart comparing neonates in case group according to phototherapy type where Group **a** represents `newborns with indirect hyperbilirubinemia and UTI confirmed by urine culture and Group **b** represents newborns with indirect hyperbilirubinemia but don’t have UTI or have pyuria. *Conventional phototherapy* is using light irradiance of 25–30 microwatts per square centimeter per nanometer (microW/cm2/nm) in the 430–490 nm band. *Intensive Phototherapy* is using light irradiance more than 30 microW/cm2/nm detected by radiometer

**Fig. 2:**
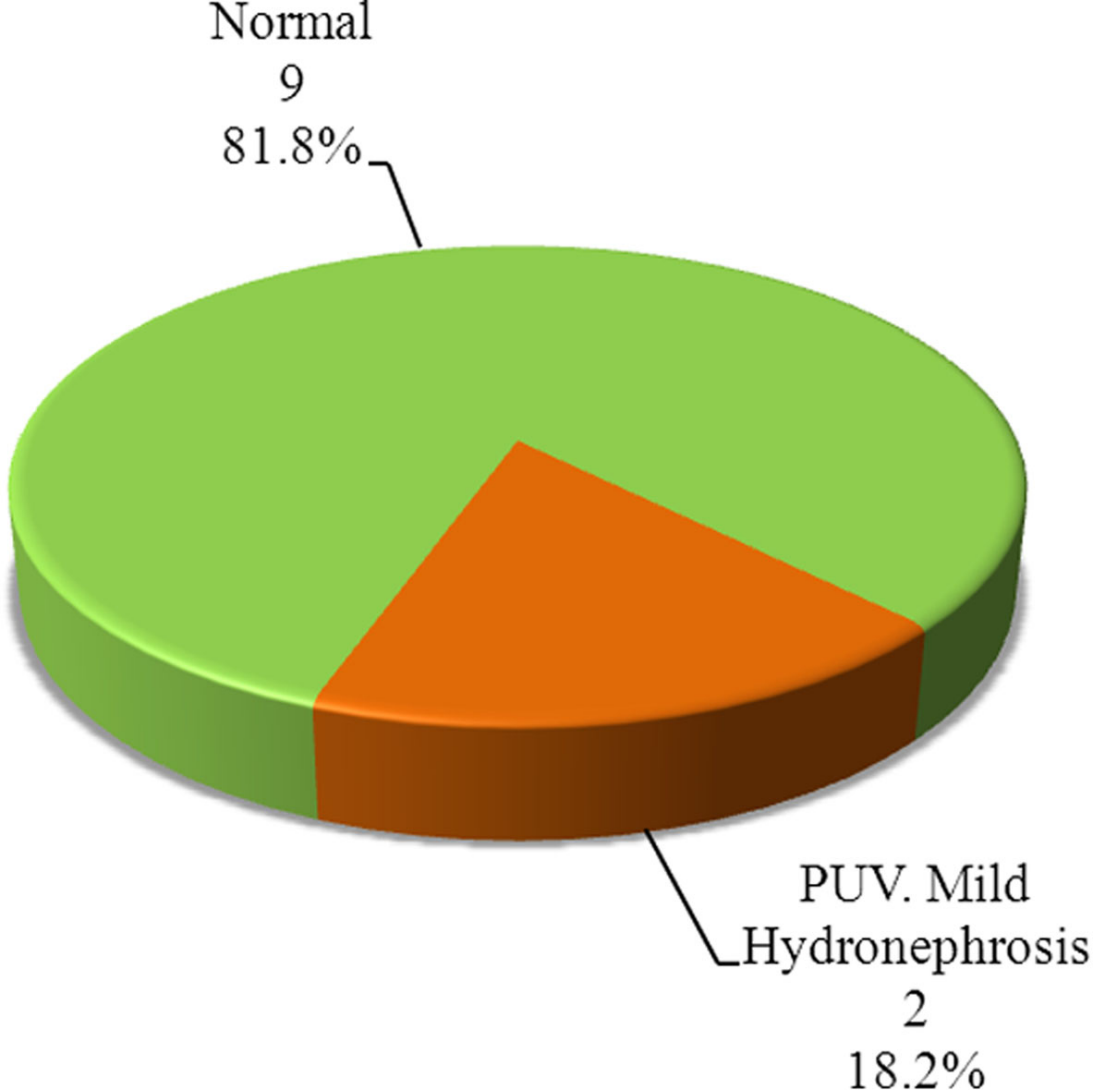
Pie chart of US kidney and pelvis results of neonates with UTI confirmed by urine culture (PUV = posterior urethral valve)

Nineteen percent of mothers of case group had history of maternal infection, 11% had UTI and 8% had PROM. (Table 3) illustrates association between results of urinary culture and some laboratory tests and clinical data. The comparative phototherapy type in case group of the UTI positive neonates (group A) vs. UTI negative neonates (group B) are illustrated in (Fig. 1). Abdominal ultrasonography (USG) with focus on kidney and bladder was performed to cases with positive culture results during hospitalisation which diagnosed two patients (18.2%) to have posterior urethral valve with mild hydronephrosis (Fig. 2).

**Table 3:** Comparison between cases confirmed to have UTI and negative cases in case group according to some clinical and laboratory variables

Variable	Urinary Tract Infection				***P*** value
	Group A (Positive)(***n*** = 11)		Group B (Negative) (***n*** = 89)		
Mean ± SD.					
**Age on admission (hours)**	120.0 ± 41.57		118.65 ± 52.97		0.674
**Weight (kg)**	3.01 ± 0.20		3.14 ± 0.36		0.255
**Total bilirubin (mg/dl)**	18.09 ± 1.45		17.87 ± 1.74		0.468
**Direct bilirubin (mg/dl)**	0.67 ± 0.33		0.68 ± 0.34		0.934
**Hemoglobin (gm/dl)**	16.05 ± 1.23		15.34 ± 1.43		0.132
**Reticulocyte count (%)**	2.64 ± 1.14		2.4 ± 1		0.454
**Urea (mg/dl)**	17.19 ± 5.13		16.67 ± 4.18		0.639
**Creatinine (mg/dl)**	0.55 ± 0.28		0.39 ± 0.20		0.081
	**No.**	%	**No.**	**%**	
**Sex**					
Male	5	45.5	38	42.7	^FE^p=1.000
Female	6	54.5	51	57.3	
**Maternal infection**					
Negative	4	36.4	77	86.5	
UTI	5	45.5	6	6.7	MCp=0.001*
PROM	2	18.2	6	6.7	
**Maternal history of chronic disease**					
Non	8	72.7	62	69.7	
Diabetes	1	9.1	9	10.1	MCp=0.001*
Hypertension	2	18.2	18	20.2	

*FE* Fisher Exact, *MC* Monte Carlo

p: p value for comparing between the studied groups

*: Statistically significant at *p* ≤ 0.05

Group A: Newborns with indirect hyperbilirubinemia and UTI confirmed by urine culture

Group B: Newborns with indirect hyperbilirubinemia but don’t have UTI or have pyuria

## Discussion

Jaundice is considered one of the most common problems in neonates. About 60% of full term infants develop jaundice [4]. Indirect hyperbilirubinemia is common and is related to a spread of physiologic and pathologic conditions. Neonates with UTI may present only with jaundice. UTI investigations have been included in routine workup of jaundice. Although investigating for UTI in neonates with significant unexplained indirect hyperbilirubinemia remains controversial [5]. Hence the aim of this study was to evaluate UTI among neonates with significant unexplained indirect hyperbilirubinemia.

This study indicated the incidence of UTI in our studied cases was 11% of jaundiced neonates while no case in control group diagnosed with UTI with significant statistical difference between 2 groups (*P* value < 0.05). In previous studies, the incidence rate of UTI in jaundiced neonates has ranged from 5.8 to 21% [6]. Ghaemi et al. [7] had a prospective study which reported UTI incidence of 5.8% in jaundiced neonates. The highest (21%) incidence of UTI was reported in a study done by omae et al. [3]. The prevalence of UTI was 16.7% in a recent study performed by Ozcan et al. [6]

In our study there was no statistical difference between incidence of UTI between males and females as six out of 11 were females while five were males. Chen et al. [8] reported similar results as UTI incidence in males was 41.7% while in females was 58.3%. Another retrospective study done by Omar et al. [4] stated that UTI in males was 59.4% while in females was 40.6% with no statistical difference. On the other hand, Cleper et al. [9] reported that the percentage of UTI in females was 6 times less than in males and the percentage was also 3 times higher in males in the study of Bilgen et al. [2] Forty-five out of 100 in case group had irrelevant family history of jaundice with no statistical difference between 2 groups.

Blood culture in all cases confirmed to have UTI by urine culture was negative. This disagrees with study done by Bahat Ozdogan et al. who found that of UTI positive jaundiced neonates, 6.2% had documented bacteremia [10]. The comparison between the positive & negative groups regarding history of maternal infections showed there was statistical significant difference between the two groups (*p*-value< 0.05).

As regards the most common isolated organisms, three out of 11 (36.4%) of positive cases were caused by *Escherichia coli* infection, 27.3% (3 out of 11) caused by acinetobacter bacilli. *E. coli* was the most common organism in studies of Chen et al. [8] and Bahat Ozdogan et al. showed that *E. coli* was the causative organism in 50% of cases [10] while Omar et al. found in their study that the most of isolated organisms were klebsiella (46.7%), and *E. coli* (37.5%) [3].

Our data showed that out of 11 cases, there were two cases diagnosed to have a posterior urethral valve (PUV) with mild hydronephrosis. The two cases had no signs of sepsis (e.g., fever, lethargy, or poor feeding), and all inflammatory markers were negative; this may attribute the bilateral hydronephrosis to mechanical obstruction by PUV rather than UTI. The guidelines of American Academy of Paediatrics (AAP) advocate doing US in all > 2-month-old infants with UTI accompanied by fever but there are no recommendations for neonates with UTI. Our study didn’t document a significant portion of USG abnormality neonates with UTI. However, in study done by Bahat Ozdogan et al., he found that there was abnormal finding in 28.1% in renal USG of jaundiced neonates confirmed to have UTI [10].

One limitation of the study is that not all the studied cases and controls have had urine culture; we performed screening for all neonates included in the study using urine analysis (including performing leukocyte esterase test (LE) and nitrite test) and microscopic examination for the presence of pyuria. This limitation was due to NICU and microbiology laboratory local policy, which restricts performing urine culture only to cases with sepsis (which was one of the exclusion criteria in the study) or cases with abnormal urine analysis or pyuria. However, obtaining urine samples using aseptic urinary catheterization technique increased the specificity of these investigations; also using aggregate urine analysis (the presence of any LE, nitrite, or pyuria > 5 WBCs/HPF) increases the sensitivity for UTI detection in infants less than 60 days of age to 99.4% as indicated in a study done by Tzimenatos L et al. [11] Also, the absence of pyuria can help in differentiation true UTI from asymptomatic bacteriuria [12].

UTI in neonatal period has many nonspecific symptoms like fever, lethargy, vomiting, anorexia, diarrhea, weight loss, changes in urine characters and jaundice. So, neonates with UTI could present early with jaundice [13]. It remains unclear how UTI is related to jaundice in neonates but there are some explanations that need further evaluating studies. Of these explanations is hepatocellular injury that may be caused directly circulating microorganisms that caused UTI or by their circulating endotoxins. On the other hand, jaundice may be the cause of UTI through making neonates more prone to infections by decreasing the bactericidal activity of their serum as reported in study done by Cisowska et al. [14].

## Conclusion

From the previous discussion, we can come to the conclusion that UTI may be one of causes of neonatal unexplained indirect hyperbilirubinemia. Also, it was found that there is a positive correlation between maternal infections and UTI in jaundiced neonates. Therefore, we can suggest that UTI investigations could be included in routine workup of neonates with unexplained indirect hyperbilirubinemia and good antenatal follow up with early treatment of maternal infections could prevent UTI in newborns.

## Supplementary Information

**Additional file 1.**

## Data Availability

All data generated or analysed during this study are included in this published article [and its supplementary information files].
